# Conductivity and Density of States of New Polyphenylquinoline

**DOI:** 10.3390/polym11060934

**Published:** 2019-05-29

**Authors:** Shamil R. Saitov, Dmitriy V. Amasev, Alexey R. Tameev, Vladimir V. Malov, Marine G. Tedoradze, Valentin M. Svetlichnyi, Lyudmila A. Myagkova, Elena N. Popova, Andrey G. Kazanskii

**Affiliations:** 1 Department of Semiconductors, Faculty of Physics, Lomonosov Moscow State University, Moscow 119991, Russia; saitsham@yandex.ru (S.R.S.); kazanski@phys.msu.ru (A.G.K.); 2 Prokhorov General Physics Institute, Russian Academy of Sciences, Moscow 119991, Russia; amoslegkie@gmail.com; 3 Frumkin Institute of Physical Chemistry and Electrochemistry, Russian Academy of Sciences, Moscow 119071, Russia; vladimir.vl.malov@gmail.com (V.V.M.); tedoradze_m@mail.ru (M.G.T.); 4 Institute of Macromolecular Compounds, Russian Academy of Sciences, Saint Petersburg 199004, Russia; valsvet@hq.macro.ru (V.M.S.); mila_myagkova@mail.ru (L.A.M.); popovaen@hq.macro.ru (E.N.P.)

**Keywords:** organic semiconductors, conducting polymers, photoconductivity, density of states, charge mobility

## Abstract

Electrical, photoelectrical, and optical properties of thin films of a new heat-resistant polyphenylquinoline synthesized using facile methods were investigated. An analysis of the obtained temperature dependences of the dark conductivity and photoconductivity indicates the hopping mechanism of conductivity over localized states arranging at the energy distance of 0.8 eV from the Fermi level located inside the band gap of the investigated material. The optical band gap of the studied material was estimated from an analysis of the spectral dependences of the photoconductivity and absorption coefficient before (1.8–1.9 eV) and after (2.0–2.2 eV) annealing at temperatures exceeding 100 °C. The Gaussian character of the distribution of the localized states of density inside the band gap near the edges of the bands was established. A mechanism of changes in the optical band gap of the investigating polymer under its annealing is proposed.

## 1. Introduction

**Scheme 1:**
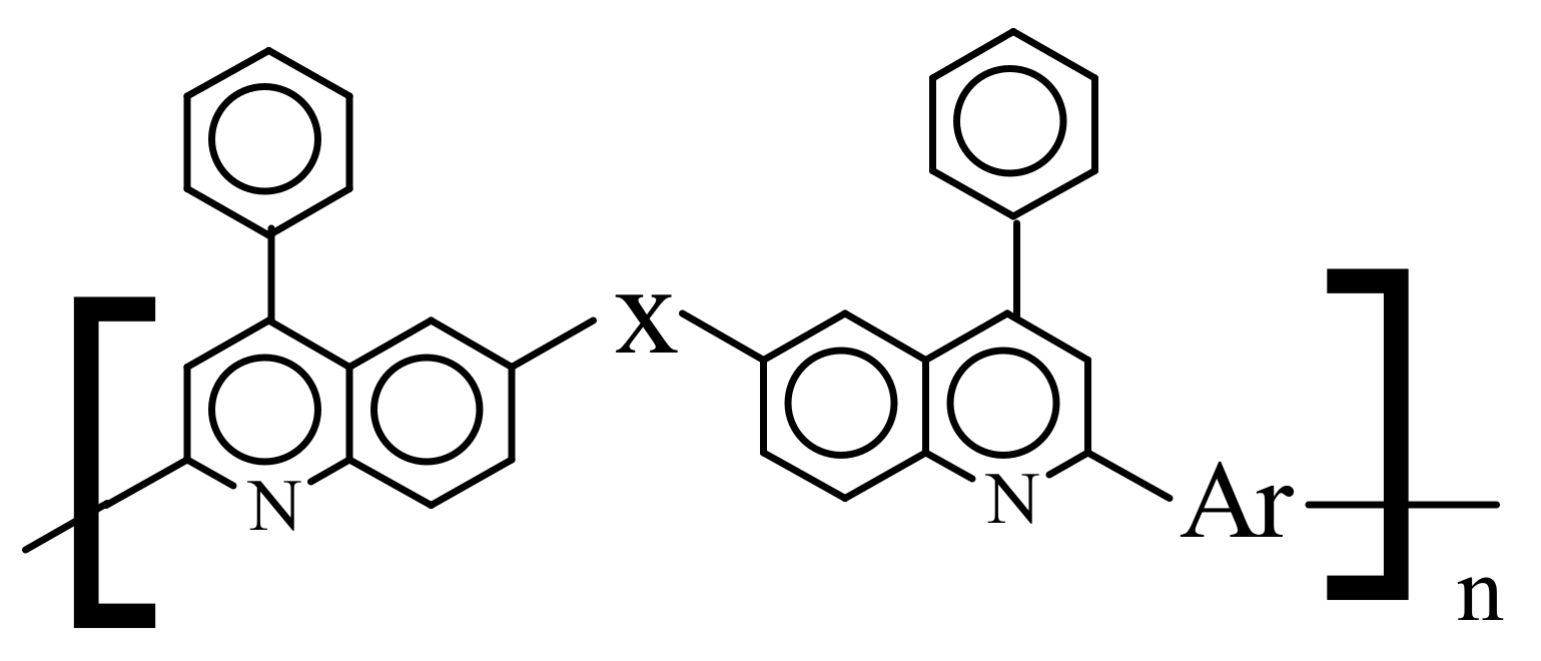
General formula of poly-2,6-phenylquinolines, where X is a bridging group, Ar is a heteroarylene moiety.

Polyquinolines have been well known for a long time as polymeric materials with high thermal and oxidative stability [1,2,3]. Such properties are also needed for conductive and photoconductive conjugated polymers developing for light-emitting diodes and photovoltaic cells [4,5,6] which certainly heat up during operation. From the point of view of photosensitivity, polymers containing both electron-donor and electron-acceptor moieties in the monomer unit are promising as photoconductive materials. From this point of view, nitrogen-containing polymers of the polyphenylquinoline class (PPQ) are of interest. The elementary unit poly-2,6-phenylquinolines (PPQs) [7] comprising an electron-accepting heterocyclic phenylquinoline (PQ) fragments separated by a bridge (X) group and a heteroarylene (Ar) electron donor fragment belong to such polymers (Scheme 1):

It was established that the monomeric unit that PPQ is an intramolecular charge transfer complex [7]. At the same time, electron donor (D) Ar-fragments and electron acceptor (A) PQ cycles form a donor–acceptor (D–A) complex. For PPQ, in which phenylamine (PA) is used as the bridging group X, forms a structure similar to triphenylamine (TPA) with the quinoline ring. A nitrogen-containing donor fragment, for example, carbazole or indolo [3,2-b] carbazole, in the UV spectra, shifts the absorption edge of PPQ (PA, Ar) significantly (up to 70 nm) to the long-wavelength region and the photosensitive and transport properties are enhanced [8]. Synthesized PPQs, which have different aromatic Ar fragments in the structure of the elementary unit, have high values of carrier mobility [9] and exhibit intensive luminescence in the visible spectral region in solution and in composites [7,10,11,12,13]. The obtained characteristics for a number of studied PPQs indicate the possibility of their use as materials for optoelectronics, in particular, polymer light-emitting diodes.

For polyphenylquinoline with a phenylamine bridging group, a balance between the hole and electron mobility of charge carriers was found. Introduction of nitrogen-containing heterocyclic structures like carbazole or indolocrbazole to the Ar fragment of the polymer chain PPQ allows to expand the class of polymers for use in field-effect transistors and photovoltaic cells [13,14,15,16].

For preparing organic light emitting diodes and photovoltaic cells, various types of electron–acceptor compounds such as perylene diimide, diketopyrrolopyrrole, benzothiadiazole are introduced into the photoactive materials [9,17,18,19,20]. 2,1,3-Benzothiadiazole (DBT), which either enhances photophysical properties of a polymer chain or serves as a dopant molecule, is of a special compound in this list [19]. In [21,22,23,24], DBT molecules were used as small additives in blend compositions with polymers to improve their optical and optoelectronic properties, and in [24,25,26,27], as a copolymerized unit in the polymer chain for modifications of light-emitting diodes and improving the efficiency of photovoltaic cells. Materials consisting of molecules containing the DBT fragment are known as organic semiconductors, having a relatively small width of the band gap equal to approximately 1.8 eV [28,29,30]. The indicated value of the band gap is optimal for creating solar cells based on organic semiconductors, including tandem solar cells.

In addition, the photoelectric properties of the material are also determined by the transport of the non-equilibrium charge carriers. Its disordered molecular structure results in a hopping mechanism. In this case, the charge carriers’ mobility temperature dependence could be described by an exponential law. Therefore, the effect of DBT molecules on the transport mechanism is also of interest.

**Figure 1:**
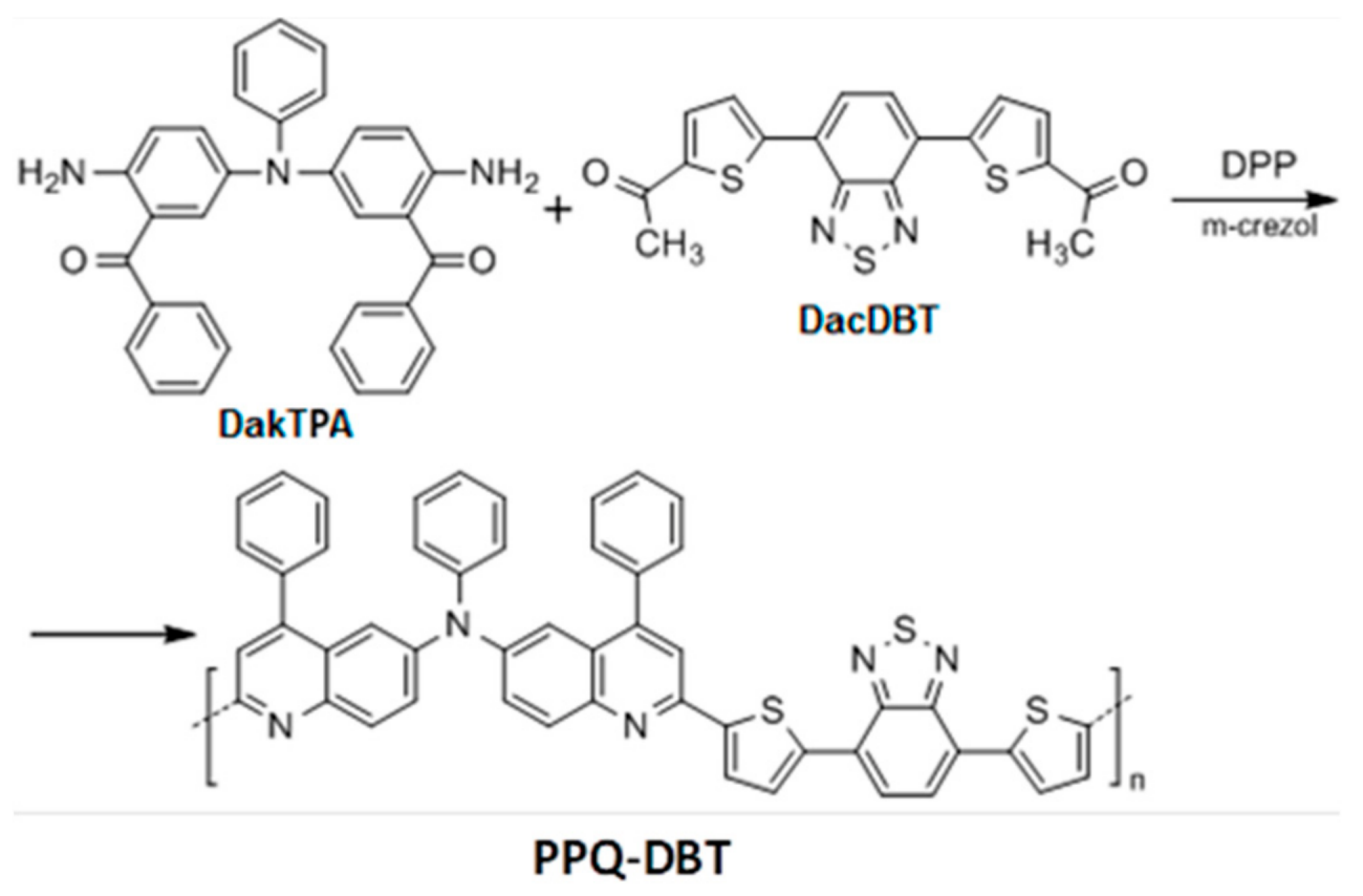
The synthesis of PPQ–DBT.

The photoelectric parameters of semiconductor materials are determined by the processes of generation of non-equilibrium charge carriers, their transport mechanisms, and recombination processes. The aim of this work was to study these processes in thin films of a new PPQ polymer, in the link of which a fragment of dithienyl-2,1,3-benzothiadiazole (DBT) is embedded. The electrical, optical, and photoelectric properties of new PPQ–DBT (Figure 1) containing triphenylamine fragment (TPA) and DBT in the main chain were investigated to determine the density distribution of electronic states in this new promising polymer. The use of this polymer when creating solar cells can lead to an increase in its temperature above room temperature. Therefore, the effect of annealing the polymer films at the temperature exceeding 100 °C was studied.

## 2. Materials and Methods

### 2.1. Synthesis of Polymer

4,7-Di(2-thienyl)-2,1,3-benzothiadiazole (TCI Europe N.V., Zwijndrecht, Belgium) 98%, m.p. 122–126 °C; diphenyl phosphate (**DPP**), 99% (Sigma-Aldrich, St. Louis, MO, USA); acetyl chloride, 98% (Sigma-Aldrich); anhydrous aluminum chloride (Sigma-Aldrich); triethylamine ≥ 99% (Sigma-Aldrich) were used as received, m-cresol 98% (Acros Organics, Geel, Belgium) was distilled prior to polymerization.

4,4′-diamino-3,3′-dibenzoyltriphenylamine (m.p. 192–194 °C) **DakTPA** was synthesized by the method described earlier [31]. New diacetyl derivative of 4,7-di(2-thienyl)-2,1,3-benzothiadiazole (m.p. 224–225 °C) **DacDBT** was synthesized by acylation of 4.7-bis (2-thienyl) -2,1,3-benzothiadiazole according to the Friedel–Crafts reaction [32].

The synthesis of poly-[6.6’-(phenylamino)bis(4-phenylquinoline)-5,5-(4’,7’-di-2-thienyl-2’,1’,3’-benzothiadiazole] (**PPQ–DBT**) performed by Friedländer reaction [33] is shown in Figure 1.

The procedure for polymerization was as follows: 3.12 g (12.5 mmol) of diphenyl phosphate, 0.242 g (0.5 mmol) of **DakTPA**, 0.192 g (0.5 mmol) of **DacDBT** and 3 mL of meta-cresol were added to the reaction flask under an inert atmosphere. The reaction was carried out for 24 h with stirring and at a temperature of 140–145 °C. The polymer was precipitated in a mixture of 75 mL of ethyl alcohol and 8 mL of triethylamine. The product was washed in a Soxhlet apparatus in a mixture of ethanol with 10% triethylamine for 24 h and dried under vacuum at a temperature of 90 °C. The yield of the product was 72%. According to elemental analysis for C_50_H_29_N_5_O_2_S_3_: Calculated, %: C–75.3; H–3.89; N–8.79; S–12.1; found, %: C–72.7; H–4.17; N–8.82: S–14.3. *M*_n_∙10^−3^ = 27.0; *M*_w_∙10^−3^ = 48.0; *M*_w_/*M*_n_ = 1.78. ^1^H NMR (DMSO-d_6_), δ(ppm): 8.43 (2H); 8.12–8.14 (8H); 8.00 (4H); 7.74–7.77 (15H) (Figure S1).

### 2.2. Characterization

Differential scanning calorimetry (DSC) was carried out using a DSC 204 F1 (Netzsch, Selb, Germany) differential scanning calorimeter to obtain the glass transition temperature (*T_g_*) of the polymer. The analysis was conducted under an inert atmosphere with samples ~4–5 mg at a scan rate of 10 °C·min^−1^ in the range from 20 to 350 °C. Reported transition was based upon the second heating. A thermobalance TG 209 F1 Libra (Netzsch) was used for thermogravimetric analysis (TGA), which was performed under inert atmosphere with samples having a weight ~2–4 mg at a scan rate of 10 °C·min^−1^. The flow rate of the inert gas passing through the sample was 40 mL·min^−1^, the flow rate of the protective gas passing through the thermocouples was 20 mL·min^−1^.

Gel chromatograms were obtained with a Waters chromatograph, a Breeze system using μ-styrogel sorbent columns (column sizes 8 × 300 mm, particle size 5 μm, pore diameters 105, 104, 103 and 102 Å). The elution mode is isocritical, the eluent is THF (flow rate 1 mL·min^−1^ at 35 °C), the concentration of solutions was 2 mg·cm^−3^.

The ^1^H NMR spectrum of PPQ–DBT was recorded on a Bruker AVANCE 400 (DMSO-d_6_) (Bruker Corp., Billerica, MA, USA).

XPS measurements were performed using OMICRON ESCA+ spectrometer (Omicron NanoTechnology, Taunusstein, Germany) with the Al-anode (the radiation energy 1486.6 eV and power 252 W). The pass energy of the analyzer was set at 20 eV, and in some cases at 10 eV to increase the resolution. To take into account the charge of the samples, the position of the XPS peaks was standardized by the C1s peak, for which binding energy, *E_b_*, was taken equal to 285.0 eV. The base pressure in the analyzer chamber was kept no higher than 8 × 10^−10^ mbar. To analyze the quantitative and qualitative composition of surface films on the samples, the spectra of C1s, O1s, N1s, F1s electrons were analyzed. The spectra were deconvoluted into components after subtraction of the background determined by the Shirley method [34]. The element ratios were calculated using integral intensities under the peaks, taking into account the photoionization cross sections of the corresponding electron shells [35]. 

The thickness of the polymer layers was measured with a KLA-Tencor D-100 Profiler (KLA Corp., Milpitas, CA, USA). Electron spectra of the layers were recorded using a Shimadzu UV-3101PC spectrophotometer (Shimadzu Corp., Kyoto, Japan).

### 2.3. Electrical Measurements

The 1 μm thickness thin film of PPQ–DBT for electrical measurements was spin-coated on a glass substrate (20 mm × 25 mm) from a PPQ–DBT solution in trifluoroacetic acid at a concentration of 10 mg/mL at room temperature in Ar atmosphere. To prepare 1 sample, the solution in a volume of 0.05 mL was cast uniformly on a 20 mm × 25 mm substrate mounted on a spin-coater. The spin-coater was turned on and rotated at 600 rpm for 7 s then accelerated during 3 s to 900 rpm and rotated for 2 min. The film obtained was dried at 70 °C for 15 min in air and at room temperature for 25 h in an Ar glovebox. In order to perform electrical measurements, aluminum contacts were deposited on the surface of the polymer film in a planar configuration using thermal spraying in a vacuum chamber. All measurements were carried out in the range of linearity of the current-voltage dependence. Measurements of the temperature dependence of the dark conductivity σ(*T*) were carried out in the vacuum of residual pressure of 10^−5^ mbar. The spectral dependences of the photoconductivity Δσ(h*ν*) and the absorption coefficient were measured at room temperature and atmosphere pressure. The Constant Photocurrent Method (CPM) [36] was used to measure the spectral dependences of the absorption coefficient α*_cpm_*.

Charge carrier mobility was measured by the charge selective CELIV (charge extraction by linearly increasing voltage) technique [37,38] via the selective extraction of each charge carrier species in a SiO_2_ inserted MIS (metal-insulator-semiconductor) structure [39]. A digital USB-oscilloscope (DL-Analog Discovery, Digilent Inc., Pullman, WA, USA) was both master pulse generator and transient current pulse recorder. The RC constant of the circuit was at least a factor of 20 smaller than the time scales of interest. The bias was swept within 100 μs and ramp, *A*, was of the order of 10^4^ V·s^−1^. For photo-CELIV measurements, samples were irradiated through the ITO side by one 100 μs, 490 nm LED flash. For the photo-CELIV measurements, the delay time was 10 μs.

Metal–Insulator–Semiconductor (MIS) devices of the structure ITO/SiO_2_/PPQ–DBT/Al were prepared. The MIS structure consisted of a charge carrier blocking SiO_2_ layer of the thickness *d_i_* = 70 nm which was preliminary deposited onto ITO-coated glass by magnetron scattering at 10^−3^ mbar, a polymer film of the thickness *d_s_* = 150 nm spin-coated from a polymer solution in trifluoroacetic acid, and a 80 nm thick Al electrode thermally evaporated under a vacuum of 10^−6^ mbar. The polymer films were dried at 70 °C for 24 h in Ar atmosphere. The measurements were carried out in a MBraun MB200MOD glovebox with Ar atmosphere at room temperature. 

A small charge extraction regime of the MIS-CELIV experiment was used and a typical signal of the transient current is shown in Figure S2. In this regime, the condition Δ*j* ≤ *j*(*0*) is fulfilled and the monopolar charge carriers are extracted as a uniform sheet of charge [40]. The corresponding small-charge transit time *t*_max_ for the sheet of carriers to reach the extracting contact defines the mobility as following:

(1) $$\mu =\frac{2d_{s}^{2}}{At_{max}^{2}}\left(1+f\right),$$

where the ratio between the geometric capacitances of the PPQ–DBT semiconductor and the SiO_2_ insulator layers *f* ≡ (*ε_s_ d_i_*)/(*ε_i_ d_s_*) is equal to ~0.3 since dielectric constant is *ε_i_* = 3.9 and *ε_s_* ≈ 2.5 for SiO_2_ and PPQ–DBT, respectively.

### 2.4. Cyclic Voltammetry (CV)

Cyclic voltammograms were recorded on a PGSTAT102 (Metrohm Autolab, Herisau, Switzerland) potentiostat using platinum electrodes at a scan rate of 20 mV·s^−1^ in a three-electrode three-compartment electrochemical cell in a glove box with a dry argon atmosphere. A 20 nm solid layer of PPQ–DBT was preliminarily deposited onto the working electrode by dip coating the Pt sheet into polymer solution in trifluoroacetic acid. Argon-saturated solution of 0.2 M of tetrabutylammonium tetrafluoborate (NBu_4_PF_6_) in acetonitrile (HPLC-grade) was used as an electrolyte. An Ag wire immersed into the electrolyte solution with the addition of 0.1 M AgNO_3_ was used as a pseudo reference electrode (Ag/Ag^+^). It was calibrated against the ferrocene/ferricenium couple (0.039 V vs Ag/Ag^+^), and its potential was recalculated to the energy scale using 4.8 eV value for Fc/Fc^+^ in acetonitrile [41]. In this case, the energy level of Ag/Ag^+^ (EAg/Ag^+^) is 4.839 eV. Taking into account the accuracy of CV experiment (±0.02 V) this value was rounded to 4.84 eV. HOMO and LUMO energy levels were determined from the oxidation (*E*_ox_) and reduction (*E*_red_) onsets of the second scan from CV data (Figure S3). The onset was determined at the crossing of a tangent to the oxidation (reduction) front extrapolated to zero current. HOMO and LUMO energy levels as well as the energy gap, *E*_g_^ec^, were calculated according to the following equations:

HOMO (eV) = −*e* (*E*_ox_ + 4.84) (2)

LUMO (eV) = −*e* (*E*_red_ + 4.84) (3)

*E*_g_^ec^ (eV) = *e* (*E*_ox_ − *E*_red_) (4)

## 3. Results and Discussion

### 3.1. Temperature Dependence of Dark Conductivity

DSC data for PPQ–DBT show that glass-transition temperature, *T*_g_, is 301 °C (Figure S4). The polymer films evaluated by TGA (Figure S5) indicate a very high thermal stability of PPQ–DBT: The decomposition of the polymer backbone begins at 550 °C. These data demonstrate well the thermal stability of PPQ–DBT.

**Figure 2:**
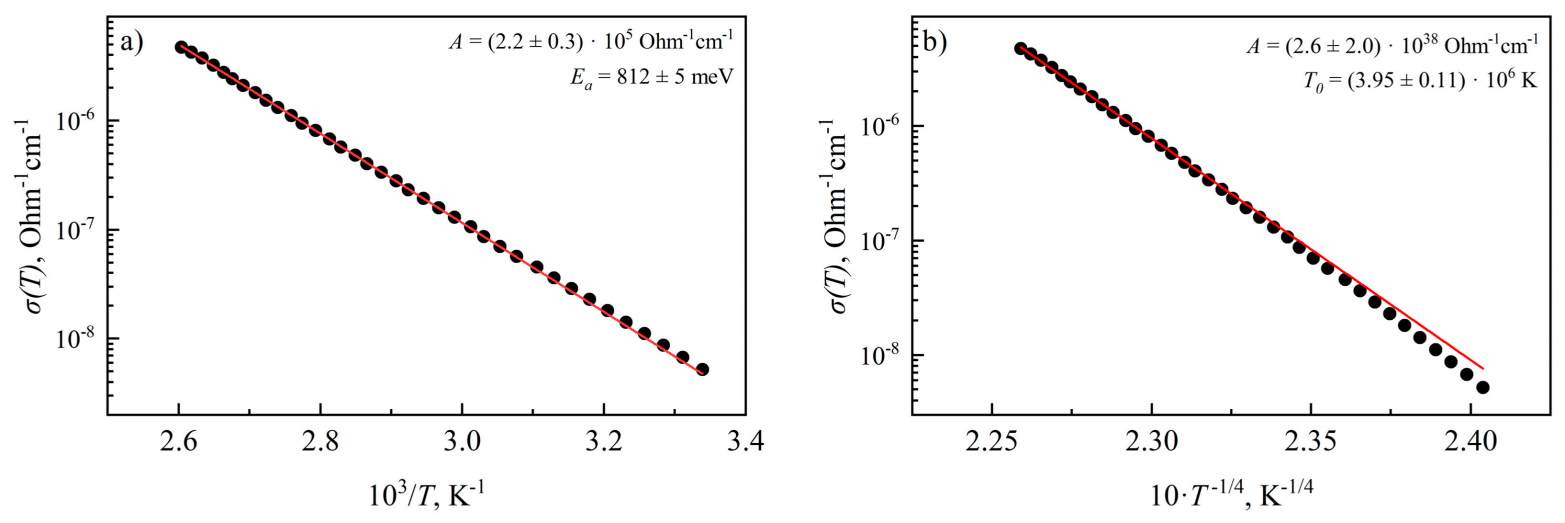
The temperature dependence of the dark conductivity in the coordinates for approximation by (**a**) activation dependence, (**b**) Mott’s law.

Dark conductivity temperature dependences investigated at the temperature range far below *T*_g_ are presented in Figure 2. As can be seen in Figure 2a, the temperature dependence of the dark conductivity can be approximated well by an Arrhenius dependence with the activation energy (*E_a_*) in the range of 0.8–0.82 eV:

(5) $$\sigma \left(T\right)=A\exp\left(-\frac{E_{a}}{k_{B}T}\right),$$

where *k_B_* isBoltzmann’s constant, *T* is temperature, and *A* is pre-exponential coefficient depending slightly on temperature (non-exponentially). The conductivity temperature dependence is determined by the temperature dependences of equilibrium charge carrier concentration and mobility. In the majority of works [42,43] devoted to the study of the charge carrier transport in polymers, it is assumed that the main mechanism is hopping transport over localized states. It is known [43] that the activation energy of charge carrier mobility, in this case, should be substantially less than the measured value of *E_a_*. Therefore, the activation energy of the conductivity temperature dependence of the sample without ellumination, apparently, should be determined mainly by the energy distance between the Fermi level and the transport level of the main charge carriers.

Note that in a number of works [44,45] it is assumed that in amorphous polymers the main transport mechanism is hopping conductivity with a variable-range hopping near the Fermi level, which is described by Mott’s law [46]:

(6) $$\sigma \left(T\right)=A\exp\left[-{\left(\frac{T_{0}}{T}\right)}^{\gamma }\right],$$

where *A* is a pre-exponential coefficient depending slightly on temperature (non-exponentially), *T*_0_ is a constant value describing the density of electronic states at the Fermi level and *γ* = (1 + *d*)^−1^. The *d* value is determined by the dimension of the space in which the studied process takes place. In our case, *d* = 3. The temperature dependence of the dark conductivity in the “Mott’s coordinates” is shown in Figure 2b. As can be seen from the Figure 2b, the obtained data of the dark conductivity temperature dependence are also well described by the Mott’s law. At the same time, in our opinion, the conductivity of the polymer is not determined by the states located near the Fermi level. Therefore, we used the technique proposed in [47] to determine the degree of conformity of the measured dependence σ*(T)* to the Mott’s law. The performed analysis gave a negative result. Thus, we assume that the equilibrium charge carrier concentration in the studied polymer determines the activation character of the σ*(T)* dependence with the activation energy *E_a_* ≈ 0.8 eV. Note that both the conductivity value and the activation energy of the conductivity temperature dependence did not change after annealing the polymer films at a temperature of 150 °C for 30 min.

### 3.2. Spectral Dependences of Photoconductivity and Optical Absorption

**Figure 3:**
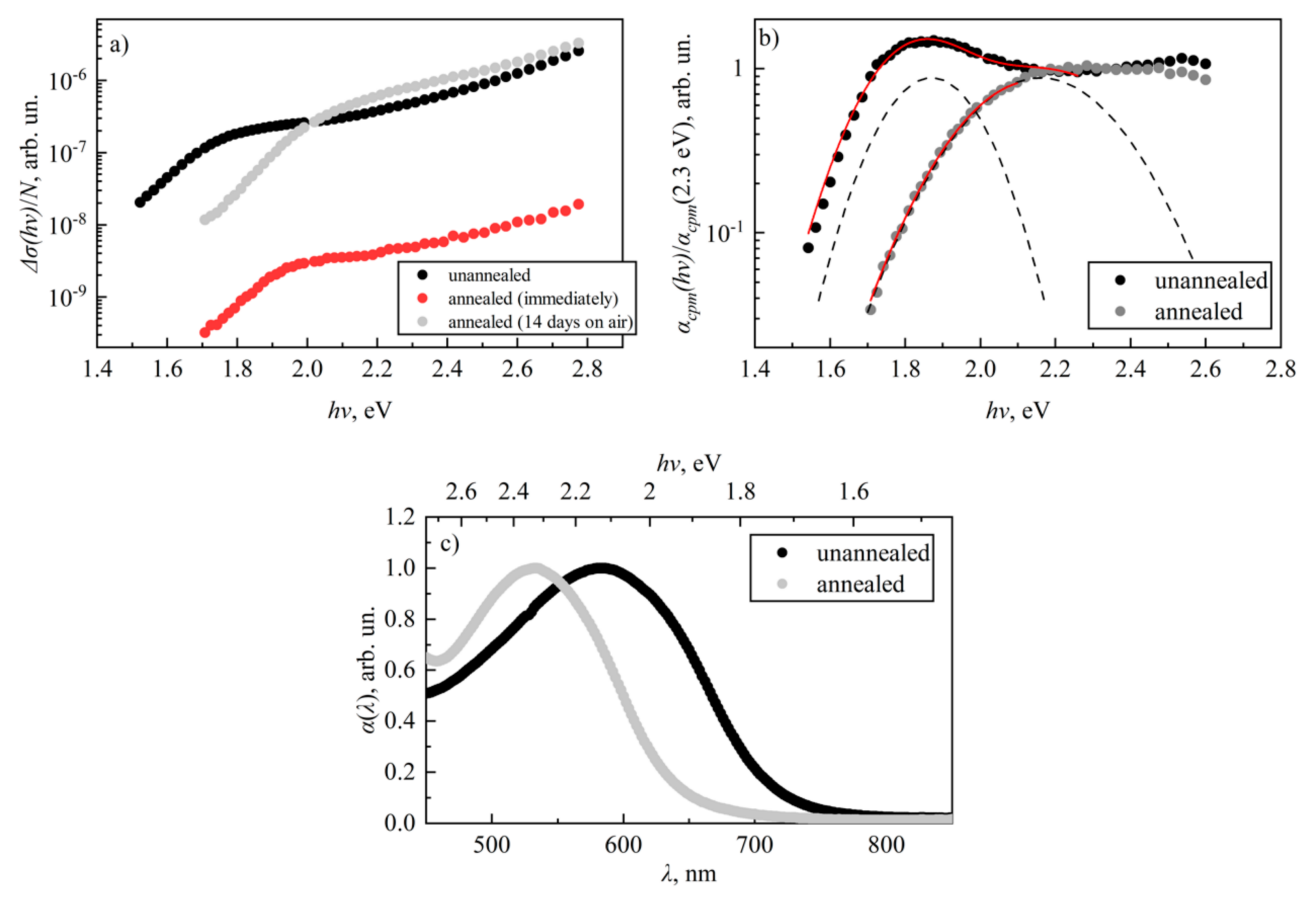
Spectral dependences of (**a**) photoconductivity Δσ, normalized to the number of incident photons *N*, (**b**) the absorption coefficient α*_cpm_*, normalized to its value at h*ν* = 2.3 eV, and (**c**) UV-Vis absorption spectra.

In the same time, annealing led to significant changes in spectral dependences of photoconductivity Δσ and absorption coefficient α*_cpm_*. Spectral dependences of Δσ(h*ν*) and α*_cpm_*(h*ν*) are shown in Figure 3a,b. These dependences were measured in the air before and after annealing of the film in the vacuum at a temperature of 150 °C for 30 min. For the annealed film, two spectra of photoconductivity are shown in Figure 3a. One of them was measured in air directly after annealing the film in vacuum, and the other after its long exposure to air (two weeks). As can be seen from Figure 3a, the exposure of the annealed sample in air does not lead to strong changes in the shape of the spectral dependence of photoconductivity, but leads to an increase in its value in the entire spectral region. Our studies have shown that a new value of photoconductivity is established after exposure to the air of annealed film for several hours. The observed effect of the air on the photoconductivity of the film annealed in the vacuum may be due to the effect of oxygen on the recombination processes in polymer films, which was noted in [48]. It should be noted that the photoconductivity spectrum shown in Figure 3a for an unannealed film was measured a few days after its creation and stay on the air.

Currently, there is no universal method for determining the optical band gap *E_g_* in organic semiconductors via the spectral dependences of the absorption coefficient. In particular, the band gap can be estimated by the position of the photoconductivity and absorption edges (the region of steep changing in slope). As can be seen in Figure 3, in range of quantum energies h*ν* ≤ 1.8–1.9 eV decreasing of photon energy leads to steep decreases in photoconductivity and absorption of unannealed samples. After annealing of samples, this range is shifting toward higher photon energies and the edges of photoconductivity and absorption reach the values of h*ν* = 2.0–2.2 eV. Therefore, it can be assumed that the optical band gap of the PPQ–DBT polymer is in the region of *E_g1_* = 1.8–1.9 eV before annealing and in the region of *E_g2_* = 2.0–2.2 eV after annealing. These values are agreed well with long-wave edges of the absorption spectra of the polymer films (Figure 3c). 

An analysis of the absorption coefficient spectral dependences presented in Figure 3b shows that in the range of h*ν* < 2.3 eV the experimental dependence obtained before the annealing of the studied polymer are approximated well by the superposition of two Gauss functions:

(7) $$\alpha_{cpm}\left(h\nu \right)\sim A\cdot \exp\left(-\frac{{\left(h\nu -E_{g1}{}^{*}\right)}^{2}}{2w_{1}^{2}}\right)+B\cdot \exp\left(-\frac{{\left(h\nu -E_{g2}{}^{*}\right)}^{2}}{2w_{2}^{2}}\right)$$

with peaks’ coordinate values *E*_*g*1_* ≈ 1.8 eV, *E*_*g*2_* ≈ 2.1 eV, and standard deviations (or peaks’ widths) equal *w*_1_ ≈ 0.12 eV, *w*_2_ ≈ 0.2 eV. Approximation parameters A and B are constant independent on *hν*. At the same time in the region of *hν* < 2.3 eV, the spectral dependence of the absorption coefficient of the studied polymer film after its annealing, is approximated well by a single Gauss function:

(8) $$\alpha_{cpm}\left(h\nu \right)\sim C\cdot \exp\left(-\frac{{\left(h\nu -E_{g}{}^{*}\right)}^{2}}{2w^{2}}\right)$$

with peaks coordinate value E_g_* ≈ 2.1 eV, standard deviation *w* ≈ 0.17 eV and approximation parameter C as a constant multiplier. The positions and shapes of the Gauss function described by the first term in the Equation (7) and the Gauss function from the Equation (8) are marked with dashed lines in the Figure 3b. It should be noted that parameters of the Gaussian in the Equation (8) (shown in Figure 3b) are close to the ones of the Gauss function represented by the second term in Equation (7).

It is known that in a disordered material α*_cpm_*(h*ν*) dependence is mainly determined by the density distribution of the “initial” and “final” electronic states involved in the optical transitions [49]. According to [50], the Gaussian distribution of the density of these electronic states in a polymer semiconductor determines the Gaussian behavior of the absorption edges of the polymer material. In this case, the position of the maximum of the Gaussian describing the absorption edge is equal to the energy distance between the positions of the maxima of the Gaussians corresponding to the density distribution of the “initial” and “final” electronic states. Thus, it can be assumed that the absorption edge of the annealed film is determined by optical transitions between two Gaussians of the density of states located at an energy distance of E_g2_^*^ ≈ 2.1 eV from each other. At the same time, the approximation of the absorption coefficient spectral dependence edge of the film before its annealing indicates that the absorption edge is formed from optical transitions between Gaussian densities of states arranged at an energy distances E_g2_^*^ ≈ 2.1 eV and E_g1_^*^ ≈ 1.8 eV from each other.

### 3.3. Density of States

**Figure 4:**
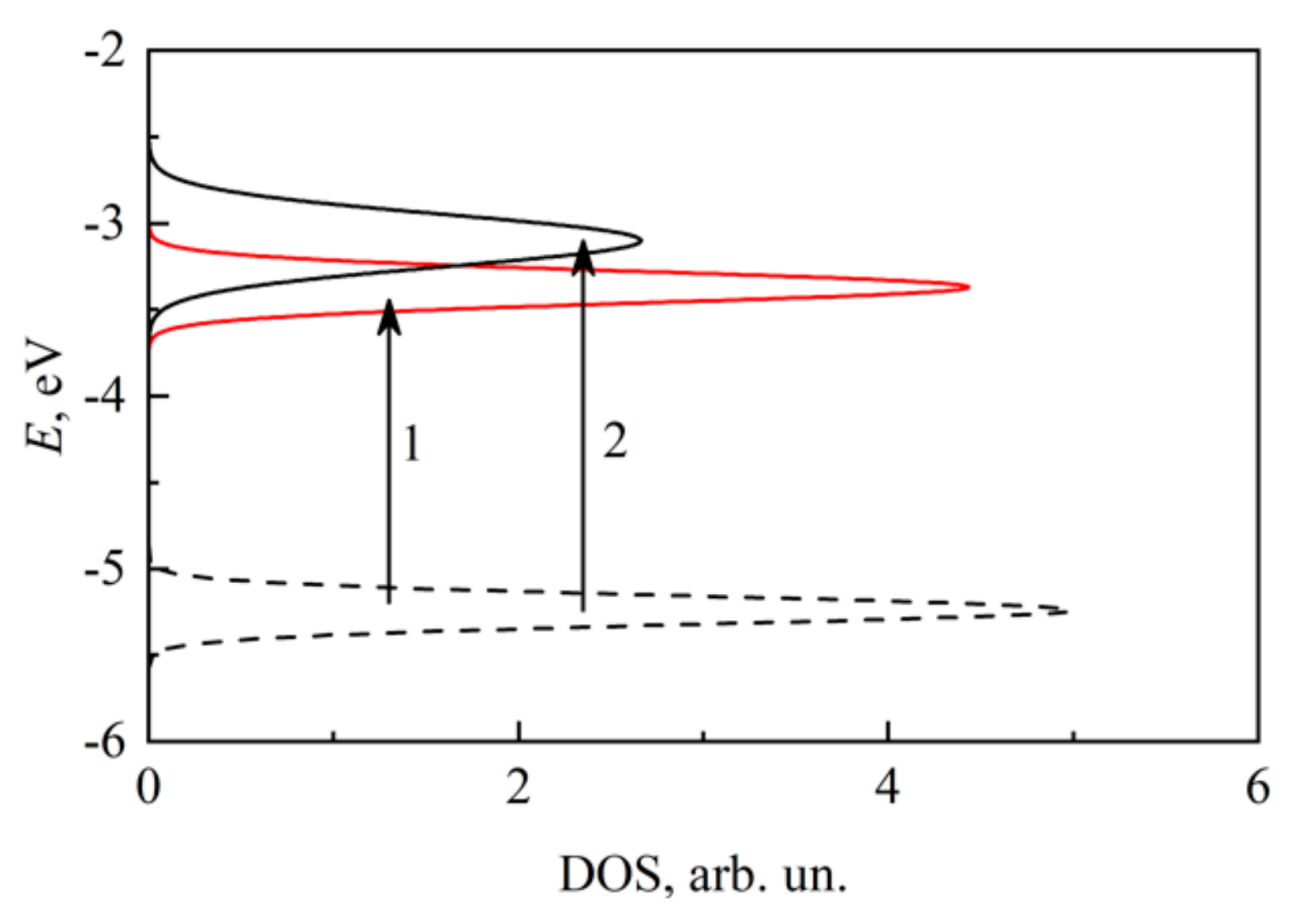
Schematic representation of the density distribution of electron energy states for molecular orbitals of PPQ–DBT fragments. Dashed black line is the HOMO DBT, solid black line–the LUMO DBT, solid red line–LUMO TPA.

It should be noted that the observed changes in the optical band gap of the investigated polymer, caused by annealing, are irreversible. We believe that the obtained changes in α*_cpm_*(h*ν*) can be explained by considering the molecular structure and electronic states energy structure (density of states) of the PPQ–DBT polymer. It can be assumed that the distribution of the electron states over the energy in the PPQ is formed by the superposition of the distribution of the electron states in the TPA and DBT fragments. This assumption is valid in the case of the weak interaction between the TPA moiety and the DBT. Figure 4 schematically shows the distribution of the electronic state density over the energy in the studied polymer that is possible in this case. The states corresponding to the electronic states of the TPA group of the polymer are indicated with red color and the states corresponding to the electronic states of the DBT moiety are indicated with black color. The dashed lines and solid lines indicate the HOMO and LUMO states, respectively. Location of initial states’ Gaussian maximum is −5.24 eV. The value estimated above corresponds to the HOMO level energy of PPQ–DBT (Figure 2a). Since the locations of these electronic states were estimated by analysis using the α*_cpm_*(h*ν*) spectra, we can estimate the density of states (DOS) for only states participating in photoconductivity.

The change of the optical band gap of the studied polymer can be explained by assuming that the annealing leads to changes in the states determining the optical absorption in the studied material. In particular, it is possible that before the film annealing, optical transition 1 between HOMO DBT and LUMO TPA and optical transition 2 between HOMO and LUMO DBT fragment marked on the Figure 4 can occur. In this case, the optical band gap is determined by the smallest energy distance – the energy distance between HOMO DBT and LUMO TPA, which is Δ*E* ~1.8 eV. If annealing reduces the probability of optical transitions 1, then the optical band gap of the annealed polymer is determined by the energy distance between HOMO and LUMO of the DBT (transition 2) and becomes Δ*E* ~2.2 eV. These Δ*E* values are close to the values of the optical band gap of the polymer film before and after annealing obtained above from the spectral dependences of α*_cpm_*.

The change of the probability of optical transitions between different states of molecules that form the PPQ–DBT as the result of annealing can be associated, for example, with the change of the mutual orientation of the TPA and DBT fragments after annealing. In this case, the irreversibility of the optical band gap changing process caused by annealing might be related to the fact that the resulting molecule structure is an equilibrium state of this molecule. Note also that the change in the probability of optical transitions between the energy states of the TPA and DBT fragments because of annealing can be associated with a desorption of the solvent used to create the polymer. Indeed, XPS analysis of the pristine and annealed polymer films revealed the rest content of fluorine was equal to 0.16 at.% and 0.07 at.%, respectively (Table S1, Figures S6 and S7). The irreversibility of this process also determines the stability of the parameters of the polymer film after its annealing.

As considered above, one of the possible causes of changes in material parameters is evaporation of a residual solvent, trifluoroacetic acid. In this case, containing dangling bonds remain radical molecules in the material. These dangling bonds are the charge carrier’s recombination centers. After a long time of placement in air atmosphere, the number of such recombination centers may decrease due to the interaction with various gases (presumably with oxygen) in the air composition. Thus, photoconductivity in annealed polymeric material after prolonged presence in air at atmospheric pressure reaches and exceeds partially the initial values for unannealed samples.

It is of importance that in both unannealed and annealed polymer films, the mobility of electrons and holes of 3.8 × 10^−4^ and 2.0 × 10^−4^ cm^2^ V^−1^ s^−1^, respectively, remains constant within the error of the measurements at room temperature. Also, an observed absence of the effect of annealing on the dark conductivity value and its temperature dependence seems to be contradictory while the optical band gap of the studied polymer irreversibly changes in result of the annealing. The obtained result for dark conductivity indicates that the transport mechanism of equilibrium charge carriers and the position of the Fermi level relative to the transport level do not change during annealing. We assume that the levels corresponding to LUMO TPA continue to participate in the equilibrium charge carriers transport after annealing the polymer film. Thus, the general picture of the distribution of electronic states for equilibrium carriers and, therefore, the mechanism of their transport does not change.

## 4. Conclusions

Using facile methods, a new heat-resistant phenylquinoline polymer was synthesized. A specific feature of the PPQ–DBT polymer is that its monomer unit consists of two types of electron donor fragments (phenylamine and thiophene) and two types of electron acceptor fragments (phenylquinoline and benzothiadiazole), which alternate sequentially in the monomer as A-D-A-D-A-D.

A study of electrical and optical properties of thin films of the polymer was carried out. Electron mobility was found to be more than hole mobility. The analysis of the temperature dependence of the dark conductivity of the polymer indicates a hopping mechanism of conduction over the localized states, arranged at an energy distance of 0.8–0.82 eV from the Fermi level located inside the band gap of the studied material. From the analysis of the absorption coefficient spectral dependence, the optical band gap of *E_g1_* ≈ 1.8–1.9 eV and *E_g2_* ≈ 2.0–2.2 eV for unannealed (as received) and annealed PPQ–DBT films, respectively, was determined. It was found that the density of electronic states inside the band gap near the edges of the bands is described by the Gauss function. We interpreted the data obtained, assuming that only LUMO and HOMO states of benzothiadiazole and phenylamine contribute to the formation of the corresponding DOS, along which the transport of electrons and holes occurs. Whether it is necessary to take into account the contribution of the phenylquinoline and thiophene fragments to the formation of DOS, additional study of proper model compounds can reveal.

The advantage of the polymer is the combination of the properties of heat resistance and photoconductivity. This is of practical significance and makes the polymer a promising material for use in development of organic photovoltaic cells, photodiodes, and light-emitting diodes. The simplicity of the synthesis of a polymer comprising several donor and acceptor fragments in its monomer unit can be a route for further development of polymers with multifunctional properties.

## Supplementary Materials

The following are available online at https://www.mdpi.com/2073-4360/11/6/934/s1, Figure S1: 1H NMR spectrum of PPQ–DBT in DMSO-d6. Figure S2: MIS-CELIV transient current of holes recorded on a load resistance of 50 Ω at 8000 V·s^−1^ ramp. Figure S3: Cyclic voltammogram of the polymer thin film on Pt sheet in 0.2M TBAPF6 in acetonitrile. The scan rate used was 20 mV·s^−1^; the oxidation (Eox) and reduction (Ered) onsets are 0.40 V and −1.30 V, respectively. Figure S4: DSC curve of the second heating cycle for PPQ–DBT at a scan rate of 10 °C/min. Figure S5: TGA curve for PPQ–DBT at a scan rate of 10 °C/min. Figure S6: Unannealed PPQ–DBT film XPS spectrum and its deconvolution into components. Figure S7: Annealed PPQ–DBT film XPS spectrum and its deconvolution into components. Table S1: The content of fluorine atoms in PPQ–DBT films obtained from XPS data.
