# Supplementary material for: Conductivity and Density of States of New Polyphenylquinoline
Source: Polymers (Basel). 2019 May 29;11(6):934. doi: 10.3390/polym11060934 (PMC6630237; doi:10.3390/polym11060934)
Supplement: Supplementary file 1 [file polymers-11-00934-s001.pdf]

Supplementary Materials

Conductivity and Density of States of New Polyphenylquinoline

Shamil R. Saitov, Dmitry V. Amasev, Alexey R. Tameev, Vladimir V. Malov, Marine G. Tedoradze, Valentin M. Svetlichnyi, Lyudmila A. Myagkova, Elena N. Popova, and Andrei G. Kazanskii

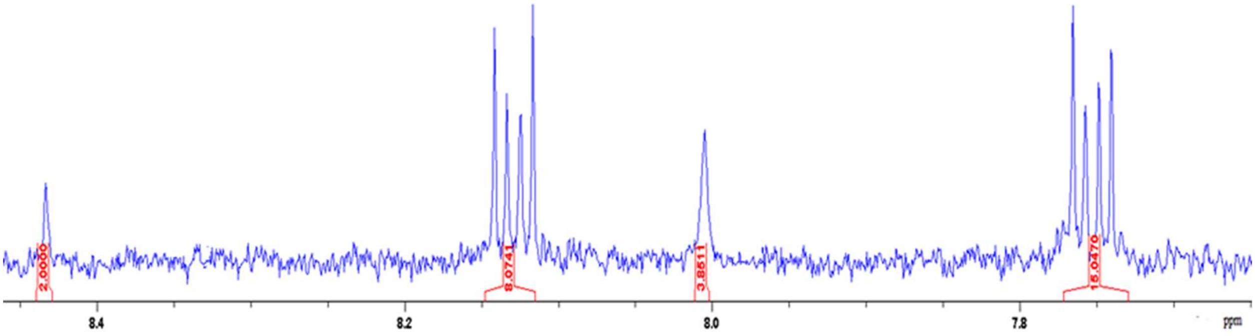

Figure S1. ^{1}H NMR spectrum of PPQ-DBT in DMSO-d_6

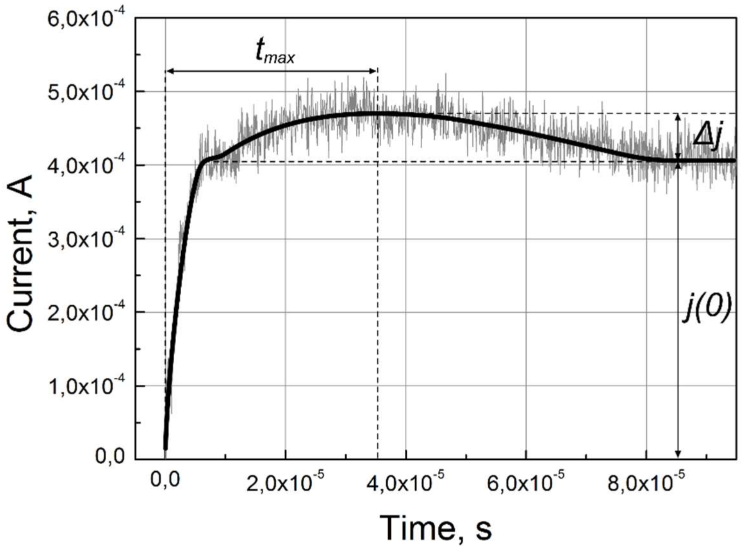

Figure S2. MIS-CELIV transient current of holes recorded on a load resistance of 50 Ω at 8000 V·s^{-1} ramp.

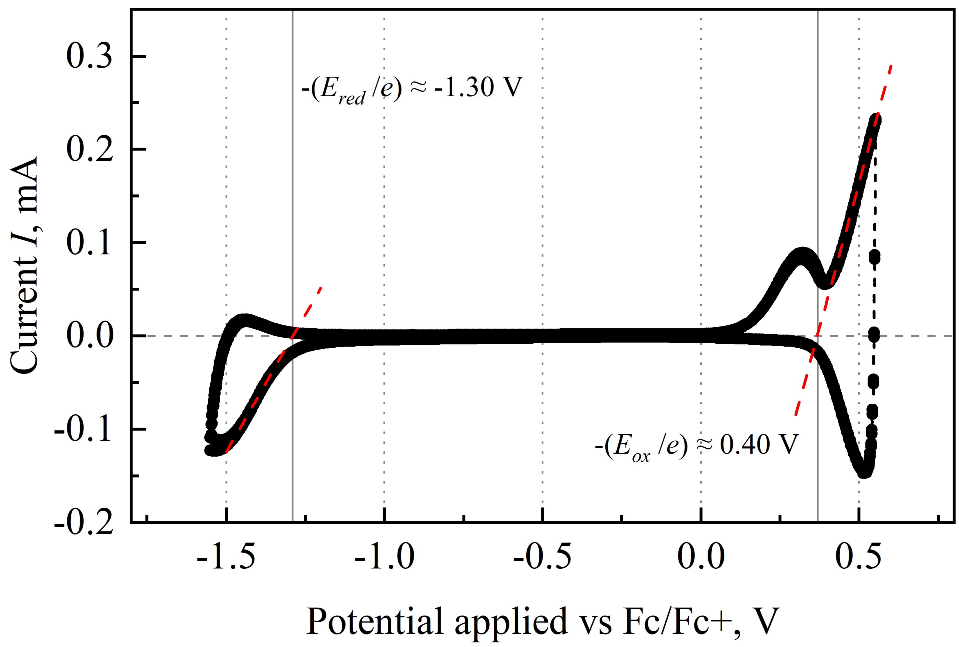

Figure S3. Cyclic voltammogram of the polymer thin film on Pt sheet in 0.2M TBAPF6 in acetonitrile. The scan rate used was 20 mV·s^{-1}; the oxidation (E_{ox}) and reduction (E_{red}) onsets are 0.40 V and -1.30 V, respectively.

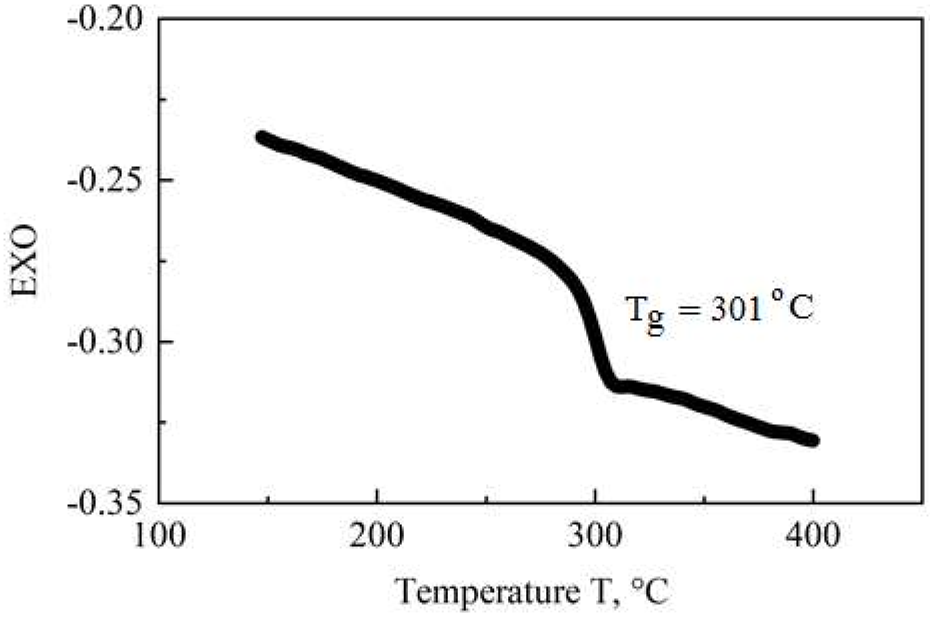

Figure S4. DSC curve of the second heating cycle for PPQ-DBT at a scan rate of 10 °C/min.

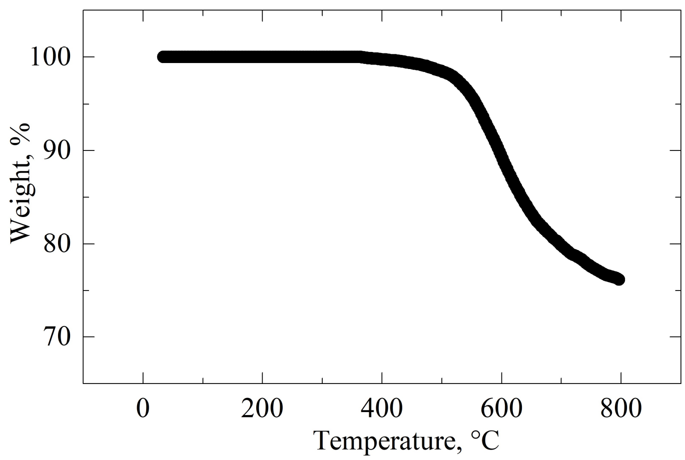

Figure S5. TGA curve for PPQ-DBT at a scan rate of 10 °C/min.

Table S1. The content of fluorine atoms in unannealed (a) and annealed (b) PPQ- DBT films obtained from XPS spectra in Figure S6 and Figure S7.

(a)

Peak name	E_B /eV	Area/cps·eV	Sens. Fact.	Norm. Area	Quant./at.%	
C1s Peak 1	285.02	314290.50	0.25	1257162.0	85.21	85.21
N1s C-N	399.84	18788.056	0.42	44733.467	3.03	3.51
N1s N-H	398.4	2994.5573	0.42	7129.8984	0.48	
O1s	532.6	108243.07	0.66	164004.65	11.12	11.12
F1s	688.9	2310.2841	1	2310.2841	0.16	0.16

(b)

Peak name	E_B /eV	Area/cps·eV	Sens. Fact.	Norm. Area	Quant./at.%	
C1s C-C/C-H	284.98	357405.66	0.25	1429622.6	50.3	88.29
C1s C-O/C-N	286.33	165411.28	0.25	661645.13	23.28	
C1s N-C=O	287.66	91734.233	0.25	366936.93	12.91	
C1s Peak 4	291.19	12785.755	0.25	51143.023	1.8	
N1s Peak 1	401.64	19038.400	0.42	45329.525	1.59	3.82
N1s Peak 2	399.7	26602.994	0.42	63340.463	2.23	
O1s Peak 1	534.61	72499.619	0.66	109847.90	3.87	7.82
O1s Peak 2	532.74	74112.898	0.66	112292.27	3.95	
F1s	690.1	1877.8423	1	1877.8423	0.07	0.07

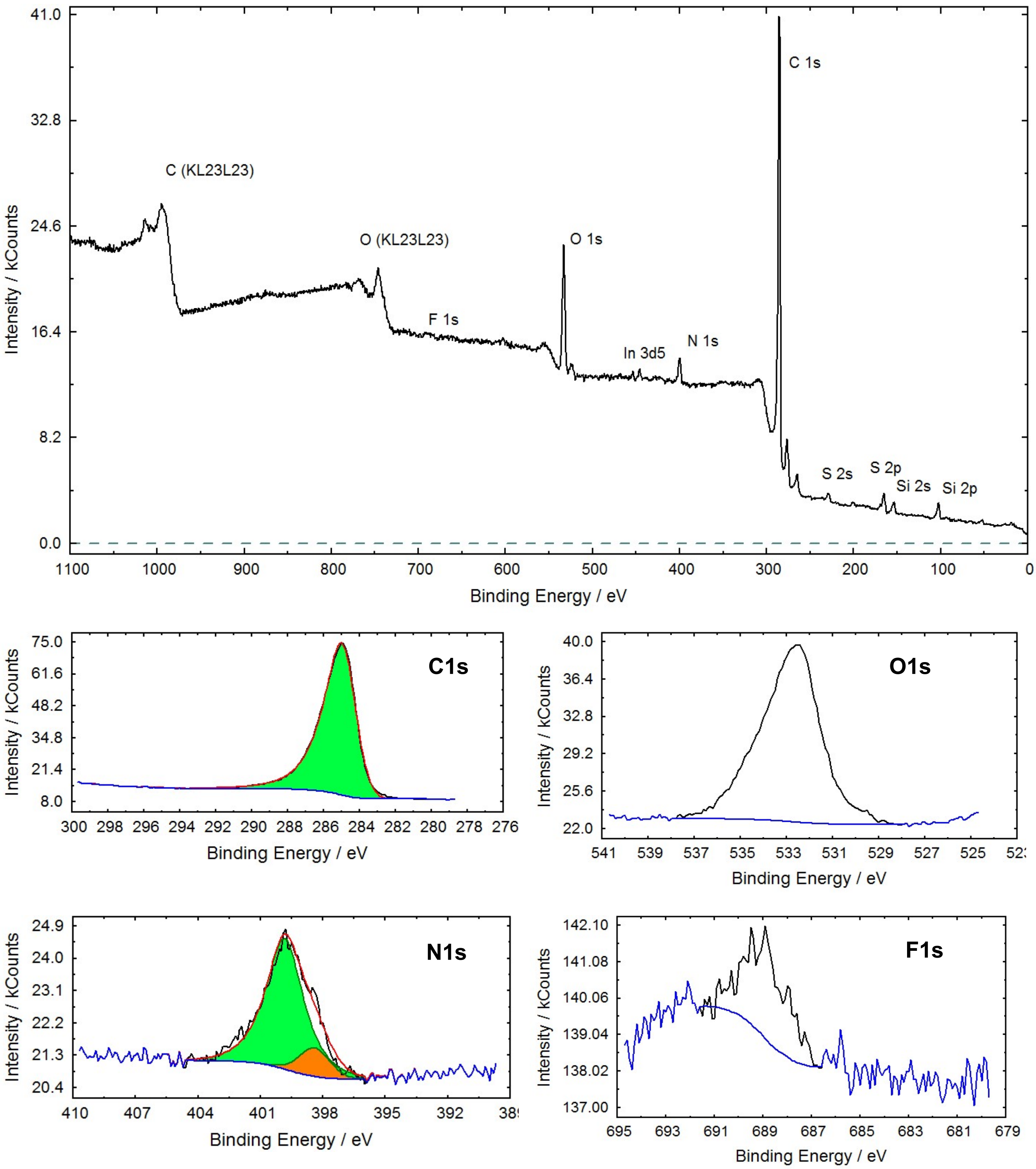

Figure S6. Unannealed PPQ- DBT film XPS spectrum and its deconvolution into components.

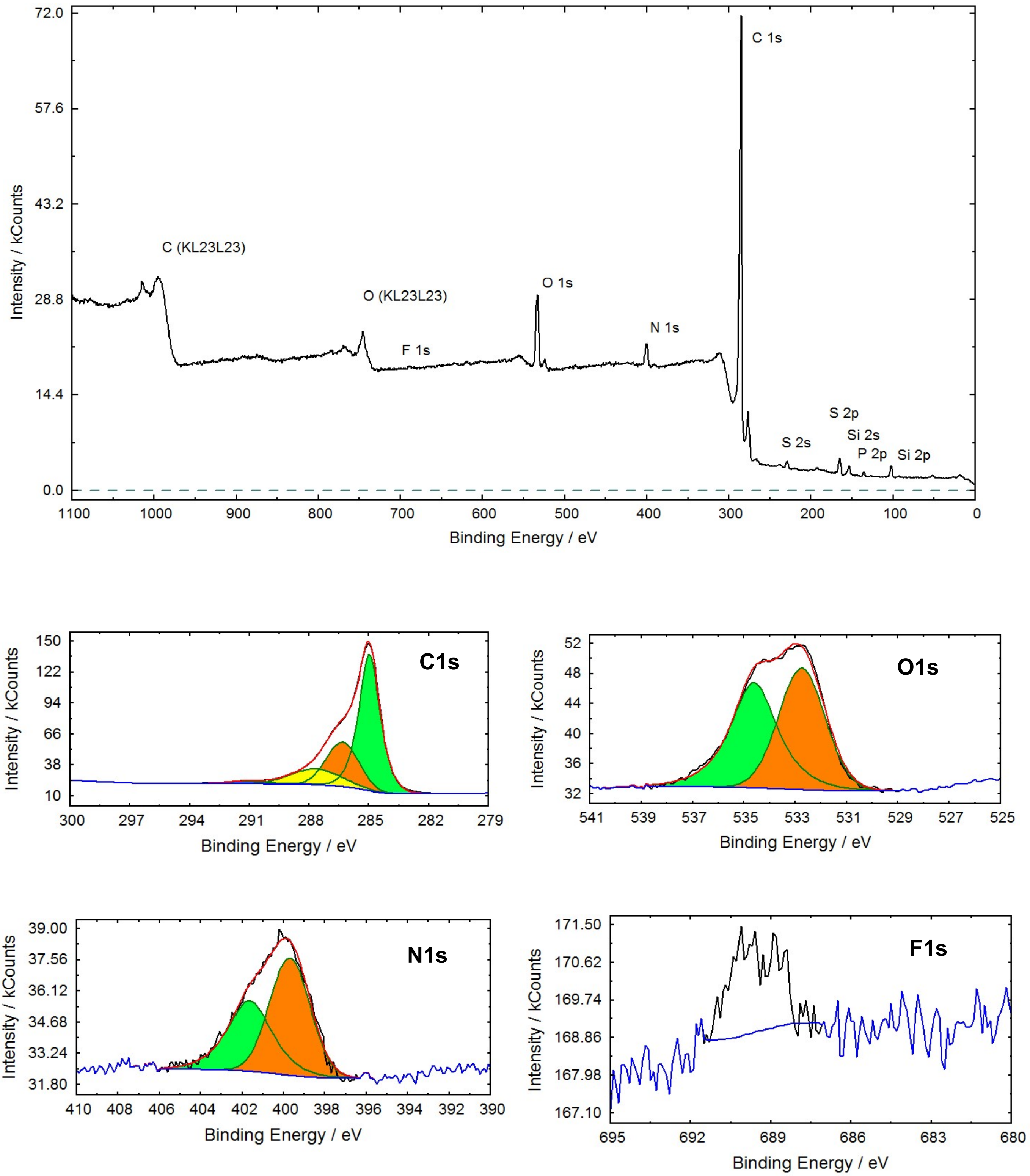

Figure S7. Annealed PPQ- DBT film XPS spectrum and its deconvolution into components.
